# Antimycobacterial and Photosynthetic Electron Transport Inhibiting Activity of Ring-Substituted 4-Arylamino-7-Chloroquinolinium Chlorides

**DOI:** 10.3390/molecules180910648

**Published:** 2013-09-02

**Authors:** Jan Otevrel, Pavel Bobal, Iveta Zadrazilova, Rodney Govender, Matus Pesko, Stanislava Keltosova, Petra Koleckarova, Petr Marsalek, Ales Imramovsky, Aidan Coffey, Jim O’Mahony, Peter Kollar, Alois Cizek, Katarina Kralova, Josef Jampilek

**Affiliations:** 1 Department of Chemical Drugs, Faculty of Pharmacy, University of Veterinary and Pharmaceutical Sciences, Palackeho 1/3, 612 42 Brno, Czech Republic; 2 Department of Infectious Diseases and Microbiology, Faculty of Veterinary Medicine, University of Veterinary and Pharmaceutical Sciences, Palackeho 1/3, 612 42 Brno, Czech Republic; 3 Department of Biological Sciences, Cork Institute of Technology, Bishopstown, Cork, Ireland; 4 Department of Environmental Ecology, Faculty of Natural Sciences, Comenius University, Mlynska dolina Ch-2, 842 15 Bratislava, Slovakia; 5 Department of Human Pharmacology and Toxicology, Faculty of Pharmacy, University of Veterinary and Pharmaceutical Sciences, Palackeho 1/3, 612 42 Brno, Czech Republic; 6 Department of Veterinary Public Health and Toxicology, Faculty of Veterinary Hygiene and Ecology, University of Veterinary and Pharmaceutical Sciences, Palackeho 1/3, 612 42 Brno, Czech Republic; 7 Institute of Organic Chemistry and Technology, Faculty of Chemical Technology, University of Pardubice, Studentska 573, 532 10 Pardubice, Czech Republic; 8 Institute of Chemistry, Faculty of Natural Sciences, Comenius University, Mlynska dolina Ch-2, 842 15 Bratislava, Slovakia

**Keywords:** 4-arylamino-7-chloroquinolines, photosynthetic electron transport inhibition, spinach chloroplasts, *in vitro* antimycobacterial activity, *in vitro* cytotoxicity, structure-activity relationships

## Abstract

In this study, a series of twenty-five ring-substituted 4-arylamino-7-chloroquinolinium chlorides were prepared and characterized. The compounds were tested for their activity related to inhibition of photosynthetic electron transport (PET) in spinach (*Spinacia oleracea* L.) chloroplasts and also primary *in vitro* screening of the synthesized compounds was performed against mycobacterial species. 4-[(2-Bromophenyl)amino]-7-chloroquinolinium chloride showed high biological activity against *M. marinum*, *M.* *kansasii*, *M. smegmatis* and 7-chloro-4-[(2-methylphenyl)amino]quinolinium chloride demonstrated noteworthy biological activity against *M. smegmatis* and *M. avium* subsp. *paratuberculosis*. The most effective compounds demonstrated quite low toxicity (LD_50_ > 20 μmol/L) against the human monocytic leukemia THP-1 cell line within preliminary *in vitro* cytotoxicity screening. The tested compounds were found to inhibit PET in photosystem II. The PET-inhibiting activity expressed by IC_50_ value of the most active compound 7-chloro-4-[(3-trifluoromethylphenyl)amino]quinolinium chloride was 27 μmol/L and PET-inhibiting activity of *ortho*-substituted compounds was significantly lower than this of *meta*- and *para*-substituted ones. The structure-activity relationships are discussed for all compounds.

## 1. Introduction

The increasing incidences of tuberculosis (TB), the number of cases of multi-drug-resistant strains of *Mycobacterium tuberculosis* (MDR-TB) and infections by non-tuberculous mycobacteria (NTM) that are connected with the increase of the number of immunocompromised patients and evolving resistance mycobacterial species to antimycobacterial chemotherapeutics make the discovery of new molecular scaffolds a priority [1,2,3,4].

The genus *Mycobacterium* consists of a closely related group of fast and slow-growing species, some of which are highly pathogenic. For example, *M. tuberculosis* causes one of the most serious human infections (tuberculosis). Difficulties should be considered while studying *M. tuberculosis*–especially a slow growth rate and the requirement for stringent containment facilities. Therefore surrogate model strains are commonly used in laboratory studies. *M.* *smegmatis* is an ideal representative of a fast-growing non-pathogenic microorganism particularly useful in studying basic cellular processes of special relevance to pathogenic mycobacteria. Additionally, *M.* *marinum* is very closely related to *M. tuberculosis* and causes TB-like infections in poikilothermic organisms, especially frogs and fish. *M. marinum* therefore, is a good surrogate model due to its lower risk for laboratory workers, genetic relatedness and similar pathology to human TB [5,6,7].

Because of the high profile of *M. tuberculosis,* the pathogenic role of other NTM in humans was overshadowed for a long time. For example, *M. kansasii*, (one of the most virulent of the NTM), causes non-tuberculous mycobacterial lung infections which are increasingly common and can be indistinguishable from tuberculosis [8]. Accordingly, it is appropriate to extend the search for novel anti mycobacterials outside of the TB complex.

Quinoline derivatives can be classified as antimycobacterial chemotherapeutics [9] and also drug design and development of other quinoline-based compounds is in progress [10,11,12,13,14,15,16,17,18]. In an excellent review by Janin hypotheses are suggested that quinoline-based derivatives can act similarly as pyrazinamide, interfere with the mycobacterial proton pump F_0_F_1_H^+^ATPase, d-alanine-d-alanine ligase, biosynthesis of amino acids or nucleic acids [10]. A number of various quinoline analogues were found as inhibitors of kinases [19]. Recently various studies were published dealing with understanding the systems for Ser/Thr and Tyr protein phosphorylation in *M. tuberculosis* and it was found that these kinases appear to regulate diverse processes including cell division and molecular transport. These facts can result in research of new antimycobacterials [20,21,22,23,24,25,26].

It was found that quinoline-like compounds exhibit herbicidal activity [15,16,17,27,28,29,30,31,32,33]. Over 50% of commercially available herbicides act by reversible binding to photosystem II (PS II), a membrane-protein complex in the thylakoid membranes, which catalyses the oxidation of water and the reduction of plastoquinone [34] and thereby inhibit photosynthesis [35,36,37].

Both pharmaceuticals and pesticides are designed to target particular biological functions, and in many cases they target similar processes or have similar molecular sites of action. For many years, virtually all pharmaceutical companies had agrochemical divisions. Leads for pharmaceuticals and pesticides often overlap, in some cases leading to similar compounds used for human health and weed management purposes. Multiple activities of various herbicides/herbicide classes show potential pharmaceutical properties, both as therapeutic agents that act through human molecular target sites and those that act on infectious agents [38,39,40]. Moreover, good correlation between antimicrobial activities and herbicidal effects was found [15,16,17,32,41,42,43,44,45,46].

A primary series of the prepared compounds contains a basic scaffold of (tyrosine)kinase inhibitors [19,47,48]. The compounds were designed as potential antimycobacterial agents with innovative effect and they were evaluated on their activity against *M. marinum*, *M. kansasii*, *M. smegmatis* and *M. avium* subsp. *paratuberculosis*. As it is known that a number of antimicrobial compounds, similarly as some of quinoline analogues/bioisosteres, display also photosynthesis inhibiting activity (bond to PS II) [15,16,17,32,33,41,42,43,44,45,46], all the prepared compounds were evaluated in relation to inhibition of photosynthetic electron transport (PET) in spinach (*Spinacia oleracea* L.) chloroplasts. Preliminary *in vitro* cytotoxicity screening of the most active derivatives was performed using the human monocytic leukemia THP-1 cell line.

## 2. Results and Discussion

### 2.1. Chemistry

**Scheme 1:**
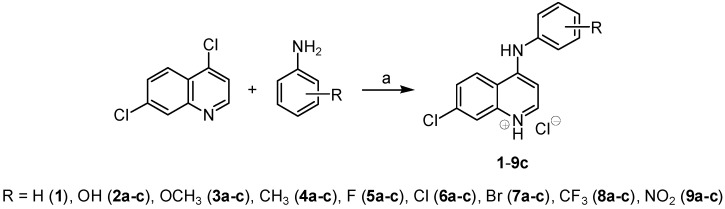
Synthesis of 4-arylamino-7-chloroquinolinium chlorides **1**–**9c**.

All the studied compounds were prepared according to Scheme 1 using a modified synthesis described by Lawrence *et al.* [49]. Reaction of 4,7-dichloroquinoline with appropriate aromatic amines in ethanol under reflux provided in quite good yields a series of twenty-five ring-substituted 4‑arylamino-7-chloroquinolinium chlorides **1**–**9c**.

The equivalent of HCl that was generated at reaction protonated the quinoline nitrogen that is much more basic than nitrogen in a spacer. p*K*_a_ values predicted using the ACD/Percepta ver. 2012 program (Advanced Chemistry Development, Inc., Toronto, ON, Canada) for unsubstituted 7-chloro-4-(phenylamino)quinoline were as follows: 5.1 ± 0.3 (quinoline nitrogen) and −0.8 ± 0.4 (spacer nitrogen). Predicted p*K*_a_ values for 7-chloro-4-[(4-hydroxyphenyl)amino]quinoline were following: 6.1 ± 0.5 (quinoline nitrogen) and −0.1 ± 0.5 (spacer nitrogen).

*Reagents and Conditions*: (**a**) ethanol, reflux.

Lipophilicity of all compounds **1**–**9c** was calculated as log *P* for the uncharged molecules using ACD/Percepta. The results are shown in Table 1. Compounds showed a wide range of lipophilicities, with log *P* values from 3.61 (**2a**, R = 2-OH) to 5.23 (**8b**, R = 3-CF_3_) within the series of ring‑substituted 4-arylamino-7-chloroquinolines. For individual substituents in the aniline part of the discussed compounds also electronic Hammett’s σ parameters were predicted using the same software; they ranged from −0.38 (**2a**, R = 2-OH) to 0.78 (**9c** R = 4-NO_2_).

### 2.2. Inhibition of Photosynthetic Electron Transport (PET) in Spinach Chloroplasts

The activity of the evaluated ring-substituted 4-arylamino-7-chloroquinolinium chlorides related to inhibition of photosynthetic electron transport (PET) in spinach (*Spinacia oleracea* L.) chloroplasts was moderate or low relative to the standard, see Table 1.

**Table 1:** Structure of ring-substituted 4-arylamino-7-chloroquinolinium chlorides **1**–**9c**, calculated values of log *P* and electronic Hammett’s σ parameters, IC_50_ [μmol/L] values related to PET inhibition in spinach chloroplasts in comparison with 3‑(3,4‑dichlorophenyl)-1,1-dimethylurea (DCMU) standard, *in vitro* antimycobacterial activity (MIC [μmol/L]) of compounds **1**–**9c** compared to isoniazid (INH), pyrazinamide (PZA), rifampicin (RIF) and ciprofloxacin (CPX) standards and *in vitro* preliminary cytotoxicity screening (LD_50_) of selected compounds.

Comp.	R	log *P ^a^*	σ *^a^*	[μmol/L]					
				PET IC_50_	MIC				LD_50_
					MM	MK	MS	MAP	
**1**	H	4.19	0	469	>879	>219	>879	>859	–
**2a**	2-OH	3.61	−0.38	ND	834	208	104	813	–
**2b**	3-OH	3.89	0.12	ND	>834	>834	>834	>813	–
**2c**	4-OH	3.71	−0.37	ND	>834	>834	>834	>813	–
**3a**	2-OCH_3_	4.66	−0.28	ND	>797	>797	**99.3**	389	–
**3b**	3-OCH_3_	4.66	0.12	238	>797	>797	>797	>778	–
**3c**	4-OCH_3_	4.66	−0.27	ND	>797	>797	>797	>778	–
**4a**	2-CH_3_	4.45	−0.17	411	>839	>839	**52.4**	**159**	>20
**4b**	3-CH_3_	4.76	−0.07	ND	ND	ND	ND	ND	–
**4c**	4-CH_3_	4.39	−0.17	ND	ND	ND	ND	ND	–
**5a**	2-F	4.58	0.06	478	>828	828	**103**	>809	–
**5b**	3-F	4.29	0.34	116	ND	ND	ND	ND	–
**5c**	4-F	4.47	0.06	370	ND	ND	ND	ND	–
**6a**	2-Cl	4.41	0.22	362	**98.6**	**49.1**	196	383	>20
**6b**	3-Cl	4.50	0.37	55	ND	ND	ND	ND	–
**6c**	4-Cl	4.18	0.23	211	ND	ND	ND	ND	–
**7a**	2-Br	4.89	0.22	251	**86.5**	**43.2**	**86.5**	>675	>20
**7b**	3-Br	5.14	0.39	89	ND	ND	ND	ND	–
**7c**	4-Br	4.89	0.23	128	ND	ND	ND	ND	–
**8a**	2-CF_3_	4.81	0.51	367	177	177	178	330	–
**8b**	3-CF_3_	5.23	0.43	**27**	ND	ND	ND	ND	>20
**8c**	4-CF_3_	5.05	0.51	**33**	ND	ND	ND	ND	>20
**9a**	2-NO_2_	5.10	0.77	132	380	380	380	743	–
**9b**	3-NO_2_	5.16	0.71	ND	ND	ND	ND	ND	–
**9c**	4-NO_2_	5.01	0.78	ND	ND	ND	ND	ND	–
**DCMU**	–	–	–	1.9	–	–	–	–	–
**INH**	–	–	–	–	467	29.2	117	>1823	–
**PZA**	–	–	–	–	–	–	–	>2031	–
**RIF**	–	–	–	–	–	–	–	>109	–
**CPX**	–	–	–	–	–	–	–	181	–

*^a^* calculated for the uncharged molecules using ACD/Percepta (Advanced Chemistry Development, Inc., Toronto, ON, Canada, 2012); MM = *M. marinum* CAMP 5644, MK = *M. kansasii* DSM 44162, MM = *M.* *smegmatis* ATCC 700084 and clinical isolate MAP = *M. avium* subsp. *paratuberculosis* CIT03. ND = not determined due to precipitation during the experiment.

**Figure 1:**
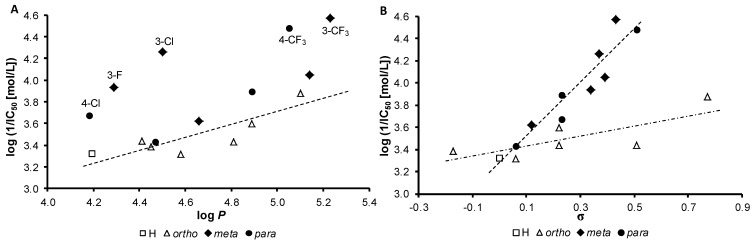
Relationships between PET inhibition log(1/IC_50_) [mol/L] in spinach chloroplasts and lipophilicity expressed as log *P* (Figure 1A) or *N*-substituent electronic Hammett’s σ parameters (Figure 1B) of selected studied compounds.

Generally compounds showed poor aqueous solubility. PET inhibition of compounds **2a**–**3a**, **3c**, **4b**, **4c** and **9b**, **9c** could not be determined due to precipitation of the compounds during the experiments. Compounds **8b** (R = 3-CF_3_) and **8c** (R = 4-CF_3_) expressed the highest PET-inhibiting activity (IC_50_ = 27 and 33 µmol/L, respectively), while compound **5a** (R = 2-F) and unsubstituted compound **1** showed the lowest PET-inhibiting activity (IC_50_ = 478 and 469 µmol/L, respectively). The PET-inhibiting activity was expressed by negative logarithm of IC_50_ value (compound concentration in mol/L causing 50% inhibition of PET). Despite the relatively low inhibitory activity of the studied compounds, correlations between log(1/IC_50_ [mol/L]) and the lipophilicity of compounds expressed as log *P* or electronic properties of individual aniline substituents expressed as Hammett’s σ parameters were performed, see Figure 1. Based on the obtained results (see Table 1, Figure 1) it can be stated that substituents in *meta*- and *para*-position are preferred from the point of view of PET-inhibiting activity compared with *ortho*-position.

The biological activity is affected by lipophilicity. In general, the PET inhibition increases with increasing lipophilicity, see Figure 1A, where the dependence of log(1/IC_50_ [mol/L]) on log *P* is illustrated. However, compounds with R = 4-Cl (**6c**), 3-F (**5b**), 3-Cl (**6b**), 4-CF_3_ (**8c**) and 3-CF_3_ (**8b**) showed substantially higher inhibitory activity than other studied compounds with comparable lipophilicity. On the other hand, PET inhibition showed linear increase with electron-withdrawing substituent for *ortho*- as well as for *meta-* and *para-*substituted derivatives (Figure 1B) and the corresponding correlations could be expressed by following equations:

*ortho-*substituted derivatives: log(1/IC_50_) = 3.371(±0.062) + 0.494(±0.168)σ r = 0.797, s = 0.131, F = 8.7, n = 7
(1)

*meta-* and *para-*substituted derivatives: log(1/IC_50_) = 3.303(±0.089) + 2.343(±0.286)σ r = 0.945, s = 0.089, F = 67.0, n = 10
(2)

The unsubstituted compound **1** is involved in both correlations. The good results of statistical analysis obtained for PET-inhibiting activity of *meta*- and *para*-substituted compounds [Equation (2)] indicate considerable effect of electronic properties of individual aniline substituents on biological activity. On the other hand, the activity of less active *ortho*-substituted derivatives was characterized by considerable variance what resulted in worse results of statistical analysis [Equation (1)]. The lower activity of *ortho*-substituted compounds could be connected with intramolecular interactions of R substituent with NH group resulting in reduced interaction of these compounds with photosynthetic proteins embedded in thylakoid membranes.

The linear course of the dependence of log(1/IC_50_) on σ indicates that for the PET-inhibiting activity not only sufficient lipophilicity (enabling easier penetration of the compounds into the lipids of photosynthetic membranes) but also sufficient electronegativity of the R substituent (affecting interactions with proteins in photosynthetic apparatus) is necessary. Compounds **8b** (R = 3-CF_3_, IC_50_ = 27 μmol/L) and **8c** (R = 4-CF_3_, IC_50_ = 33 μmol/L) were the most active compounds from the series, and this result can indicate that PET inhibition can be associated with favourable interaction of the trifluoromethyl moiety with photosynthetic proteins. A strong dependence of PET-inhibiting activity on σ was also found for 2-benzylsulphanylbenzimidazoles [50].

For specification of the site of action of certain PS II inhibitor an artificial electron donor 1,5‑diphenylcarbazide (DPC) acting in Z^●^/D^●^ intermediate [51] that is situated on the donor side of PS II can be used. If addition of DPC results in complete PET restoration in chloroplasts activity of which was suppressed by an inhibitor, the site of inhibitory action of this inhibitor is situated on the donor side of PS II in the section between oxygen evolving complex and Z^●^/D^●^ intermediate. On the other hand, if PET was interrupted on the acceptor side of PS II, despite supply of electrons by DPC, the electron flow cannot be restored. Because addition of DPC to chloroplasts activity of which was inhibited by the studied compounds (up to 30% of the control) restored PET only partially (at the most 74%–85% of the control) it can be concluded, that the sites of action of these inhibitors are situated on both sides of PS II. That means that the studied compounds act besides of donor side of PS II also on its acceptor side, namely in the section between the core of PS II (P680) and the secondary plastoquinone acceptor Q_B_. The site of action situated on the donor side of PS II was found also for 2-alkylthio-6-R-benzothiazoles (R = 6‑formamido-, 6-acetamido-, and 6-benzoylamino-) [52], anilides of 2-alkylpyridine-4-carboxylic acids acting in intermediates Z^●^/D^●^ [53], 5-bromo-*N*-phenylbenzamides [54] and 2-alkylsulphanyl-4-pyridinecarbothioamides acting in the D^●^ intermediate [55], while anilides of 2-alkyl-substituted 4‑pyridinecarboxylic acid with hydroxy substituent in the anilide part of the compound [53] as well as some anilides of *N-*benzylpyrazine-2-carboxamides [56] inhibited PET also between P680 and plastoquinone Q_B_ occurring on the acceptor side of PS II.

**Figure 2:**
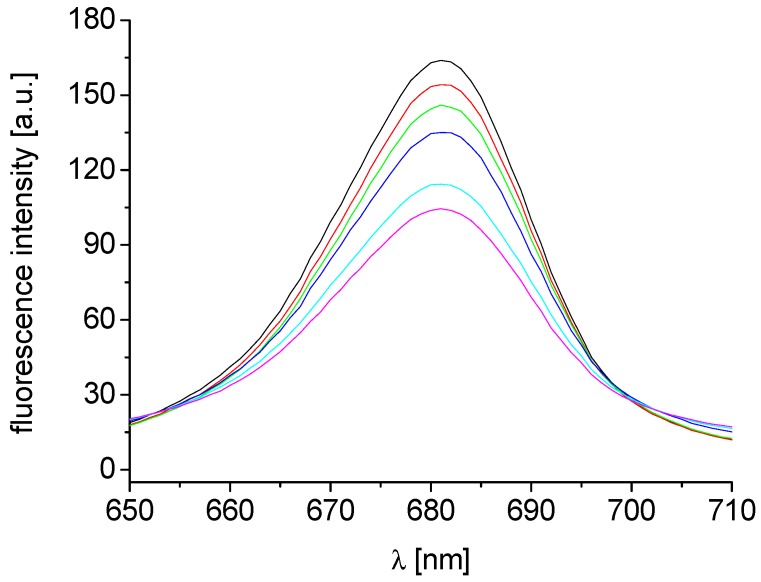
Fluorescence emission spectra of chlorophyll *a* in untreated spinach chloroplasts in presence of compound **8b**: 0, 0.06, 0.12, 0.24, 0.48 and 0.72 mmol/L (curves from top to bottom; λ_ex_ = 436 nm). Chlorophyll concentration in chloroplast suspension was constant, 10 mg/L.

The effects of the studied compounds on the photosynthetic apparatus of spinach chloroplasts were investigated by studying chlorophyll *a* (Chl*a*) fluorescence using chloroplast suspension with constant chlorophyll concentration 10 mg/L. The fluorescence emission band at λ = 686 nm belongs to the pigment-protein complexes in PS II [57] and the perturbation of chlorophyll *a*-protein complexes in the thylakoid membrane caused by PET inhibitors is reflected in fluorescence decrease. As in the presence of studied compounds the intensity of this emission band showed gradual decrease with increasing concentration of **8b**, PS II could be suggested as the site of action of this compound, see Figure 2, and similar effect was observed also with other studied compounds. A decline of Chl*a* fluorescence at λ = 686 nm was observed previously in the presence of substituted carboxamides/anilides [41,43,45,46,53,54,55,56,58], quinoline derivatives [33] and *N*-substituted 2‑aminobenzothiazoles [44,52].

### 2.3. In Vitro Antimycobacterial Evaluation

The evaluation of the *in vitro* antimycobacterial activity of all the compounds was performed against *Mycobacterium marinum*, *M. kansasii* and *M. smegmatis* as model species for screening of prospective antimycobacterial drugs to control mycobacterial diseases. A clinical isolate of *M. avium* subsp. *paratuberculosis*, which causes gastrointestinal diseases and is resistant to standard antituberculotics [59], was also involved in the screening. Isoniazid was chosen as a standard for all tested strains, nevertheless as it was observed that isoniazid is inactive against the clinical isolate, pyrazinamide and rifampicin (as first-line antituberculotic drugs) and ciprofloxacin (as an alternative antituberculotic/antimycobacterial drug) were also used as standards for estimation of the resistance of this clinical isolate. Most of compounds showed only moderate or no activity, see Table 1, only *ortho*‑substituted derivatives demonstrated some antimycobacterial effect, the rest of compounds displayed problematic aqueous solubility. 4-[(2-Bromophenyl)amino]-7-chloroquinolinium chloride (**7a**) showed the highest biological activity against *M. marinum* and *M. kansasii*, and it was also active against *M. smegmatis*. 7-Chloro-4-[(2-methylphenyl)amino] quinolinium chloride (**4a**) demonstrated the highest biological activity against *M. smegmatis* and *M. avium* subsp. *paratuberculosis*. Both compounds showed comparable or higher activity than the standard isoniazid. Compound **4a** showed even higher activity than the standard ciprofloxacin in case of the clinical isolate *M. avium* subsp. *paratuberculosis*.

**Figure 3:**
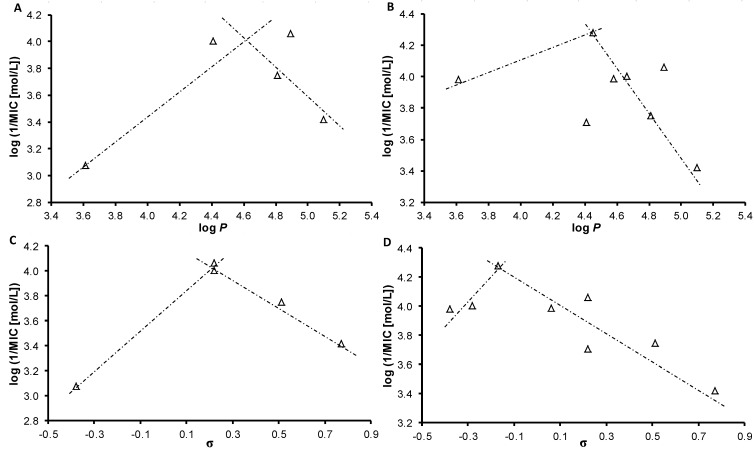
Dependences of *in vitro* antimycobacterial activity against *M. marinum* log(1/MIC [mol/L]) on lipophilicity (Figure 3A) and on *N*-substituent electronic Hammett’s σ parameters (Figure 3C) or *in vitro* activity against *M. smegmatis* log(1/MIC [mol/L]) on lipophilicity (Figure 3B) and on *N*-substituent electronic Hammett’s σ parameters (Figure 3D) of studied ring-substituted 4-arylamino-7-chloroquinolinium chlorides.

Although the number of compounds demonstrating antimycobacterial activity is limited, correlations between log(1/MIC [mol/L]) and electronic properties of individual substituents on aniline ring expressed as Hammett’s σ parameters or the lipophilicity of compounds expressed as log *P* can be found, see Figure 3. According to the results (see Table 1), it can be generally concluded that activity is significantly influenced by electronic properties and lipophilicity. The optimal range of lipophilicity, log *P* = 4.41–4.89 (Figure 3A,B), facilitates permeation through hydrophobic mycobacterial wall, while electronic properties can influence the potential of binding of the arylamino quinoline scaffold to possible binding sites [10]. The dependencies between activity and electronic Hammett’s σ parameters for all the compounds were observed as bilinear with the σ optimum ca. 0.22 for *M. marinum* and *M. kansasii*, and with the σ optimum ca. −0.17 for *M. smegmatis* and *M. avium* subsp. *paratuberculosis*, see Figure 3C,D. This fact may correspond with different site of action/binding site in *M. marinum*/*M.* *kansasii* and in *M. smegmatis*/*M. avium* subsp. *paratuberculosis*, because *ortho*‑substitution influences electron distribution in the whole aromatic conjugated system. Moreover, the antimycobacterial activity of these *ortho*-derivatives is evidently related to twist conformation of the whole arylaminoquinoline scaffold, because the proximity of the *ortho*-substituent to the quinoline nucleus on the aniline ring led to the twist of the aniline ring plain towards the quinoline nucleus, *i.e.* the whole cyclic system is not planar. Similar SAR non-planar requirements can be also found, for example, for clonidine, phenamates, phenacs, *etc.* [9].

### 2.4. *In vitro* Cytotoxicity Assay

The preliminary *in vitro* screening of cytotoxicity of the selected most effective compounds **4a**, **6a**, **7a**, **8b** and **8c** was performed using the human monocytic leukemia THP-1 cell line. The cytotoxicity was evaluated as the LD_50_ value (LD_50_ ‑ lethal dose to 50% of the cell population ‑ see Table 1). In the past several works were published, where the toxicity of tested compounds (including antibacterial agents [16,44,45,46]) was assessed on THP-1 cells. Due to low aqueous solubility of the studied compounds their utilizable concentration range in the cytotoxicity test was significantly limited. The highest dose of all tested compounds in the medium (20 μmol/L) did not lead to significant lethal effect on THP-1 cells. All the evaluated compounds demonstrated low toxicity in the highest available concentration against the human monocytic leukemia THP-1 cell line with LD_50_ > 20 μmol/L. As LD_50_ values of oxaliplatin and camptothecin assessed in this line formerly were by one or two orders lower (1.7 ± 0.64 μmol/L and 0.16 ± 0.07 μmol/L, respectively), the discussed substances can be considered as non-toxic agents for subsequent design of novel antimycobacterial agents.

## 3. Experimental

### 3.1. General

All reagents were purchased from Sigma-Aldrich (Munich, Germany). The plates were illuminated under UV (254 nm) and evaluated in iodine vapour. The melting points were determined on Kofler hot-plate apparatus HMK (Franz Kustner Nacht KG, Dresden, Germany) and are uncorrected. Infrared (IR) spectra were recorded on a Smart MIRacle™ ATR ZnSe for Nicolet™ Impact 410 FT-IR spectrometer (Thermo Electron Corporation, West Palm Beach, FL, USA). The spectra were obtained by accumulation of 256 scans with 2 cm^−1^ resolution in the region of 4000–600 cm^−1^. All ^1^H- and ^13^C‑NMR spectra were recorded on a Bruker Avance III 400 MHz FT‑NMR spectrometer (400 MHz for ^1^H and 100 MHz for ^13^C, Bruker Comp., Karlsruhe, Germany). Chemicals shifts (δ) are reported in ppm. Proton chemical shifts in DMSO-*d*_6_ are related to the middle of the solvent multiplet (δ = 2.50). ^13^C-NMR spectra were measured using APT pulse sequences. Carbon chemical shifts are referenced to the middle of the solvent multiplet (δ = 39.5 in DMSO-*d*_6_). Mass spectra were measured using a LTQ Orbitrap Hybrid Mass Spectrometer (Thermo Electron Corporation, West Palm Beach, FL, USA) with direct injection into an APCI source (400 °C) in the positive mode.

HPLC monitoring analyses were performed on Thermo Scientific UHPLC Accela 1250 system connected to a Thermo Scientific TSQ Quantum Access MAX Triple Quadrupole Instrument (Thermo, San Jose, CA, USA) equipped with heated electrospray ionization (HESI-II) probe. A Thermo Scientific Hypersil C_18_ (2.1 mm × 50 mm, 1.9 μm) column was used at a constant flow rate of 300 μL/min. Mobile phase consisted of water containing 0.1% formic acid (*v/v*) (solvent A) and acetonitrile containing 0.1% formic acid (solvent B). The gradient used was: 0–10 min from 20 to 95% B; 10‑11 min from 95 to 20% B and 11–11.5 min held at 20% B in order for the column to re‑equilibrate before the next injection. The full loop injection volume was set at 10 μL. The heated electrospray ionization was operated in the positive-ion mode under the following conditions: Capillary Temperature: 325.0 °C; Vaporizer Temperature 300.0 °C; Sheath Gas Pressure 35.0 psi; Auxiliary (drying) gas 10 a.u.; Spray Voltage 3300 V. Formic acid was purchased from Sigma-Aldrich (Munich, Germany). All solvents were residual analysis purity (Chromservis, Prague, Czech Republic).

### 3.2. Synthesis

General Procedure for synthesis of 4-arylamino-7-chloroquinolinium chlorides **1**–**9c**

The appropriate aromatic amine (5 mmol) was added at once to solution of 4,7-dichloroquinoline (5 mmol) in 40 mL of 96% ethanol at 25 °C. The reaction was heated and kept under reflux until HPLC analysis revealed complete disappearance of the starting materials. Reaction media was cooled to ambient temperature and formed precipitate was separated by filtration and triturated with 40 mL of fresh hot 96% ethanol. Suspension was cooled again, filtered and solid was successively washed with 96% ethanol and dichloromethane. Yellow to orange product was dried in desiccator under reduced pressure.

*7-Chloro-4-(phenylamino)quinolinium chloride* (**1**). Yield 87%; Mp. 274–276 °C; IR (Zn/Se ATR, cm^−1^): 2638w, 1609w, 1576w, 1533w, 1448w, 1361w, 1205w, 894w, 804m, 739m, 687s; ^1^H-NMR (DMSO-*d*_6_), δ: 11.28 (br. s, 1H), 8.93 (d, *J =* 9.0 Hz, 1H), 8.51 (d, *J =* 7.0 Hz, 1H), 8.20 (d, *J =* 2.0 Hz, 1H), 7.86 (dd, *J =* 9.0 Hz, *J =* 2.3 Hz, 1H), 7.60–7.56 (m, 2H), 7.51–7.48 (m, 2H), 7.46‑7.42 (m, 1H), 6.77 (d, *J =* 7.0 Hz, 1H); ^13^C-NMR APT (DMSO-*d*_6_), δ: 154.89, 143.32, 139.08, 138.36, 136.97, 129.97, 127.62, 127.32, 126.25, 125.47, 119.17, 115.94, 100.22; HR-MS: for C_15_H_12_ClN_2_^+^ [M+H]^+^ calculated 255.0684 *m/z*, found 255.0680 *m/z*.

*7-Chloro-4-[(2-hydroxyphenyl)amino]quinolinium chloride* (**2a**) [60]. Yield 90%; Mp. 269–270 °C; IR (Zn/Se ATR, cm^−1^): 3156w, 2774w, 1609w, 1585m, 1544m, 1442s, 1367m, 1233w, 1202m, 1157w, 882w, 844w, 797m, 741s, 655s; ^1^H-NMR (DMSO-*d*_6_), δ: 10.99 (br. s, 1H), 10.32 (br. s, 1H), 8.91 (d, *J* *=* 9.0 Hz, 1H), 8.48 (d, *J =* 7.0 Hz, 1H), 8.19 (s, 1H), 7.8 (d, *J =* 8.8 Hz, 1H) 7.28–7.31 (m, 2H), 7.17 (d, *J =* 8.0 Hz, 1H), 6.94 (t, *J =* 7.4 Hz, 1H), 6.30 (d, *J =* 6.8 Hz, 1H); ^13^C-NMR APT (DMSO-*d*_6_), δ: 155.49, 153.01, 142.80, 139.06, 138.37, 129.65, 128.31, 127.29, 126.55, 123.51, 119.98, 119.23, 117.40, 115.78, 101.16; HR-MS: for C_15_H_12_ClN_2_O^+^ [M+H]^+^ calculated 271.0633 *m/z*, found 271.0637 *m/z*.

7*-Chloro-4-[(3-hydroxyphenyl)amino]quinolinium chloride* (**2b**) [49]. Yield 48%; Mp. 298–299 °C; IR (Zn/Se ATR, cm^−1^): 3160w, 2805w, 1610m, 1577s, 1539m, 1497w, 1448s, 1409w, 1373w, 1297w, 1210m, 1160w, 1095w, 876w, 848w, 806m, 770m, 669s; ^1^H-NMR (DMSO-*d*_6_), δ: 11.15 (s, 1H), 10.05 (br. s, 1H), 8.88 (d, *J =* 9.3 Hz, 1H), 8.51 (d, *J =* 7.0 Hz, 1H), 8.17 (d, *J =* 2.0 Hz, 1H), 7.82 (dd, *J* *=* 9.0 Hz, *J =* 1.8 Hz, 1H), 7.34 (t, *J =* 8.0 Hz, 1H), 6.89–6.79 (m, 4H); ^13^C-NMR APT (DMSO-*d*_6_), δ: 158.78, 154.86, 143.19, 139.04, 138.34, 137.84, 130.67, 127.25, 126.25, 119.11, 115.89, 115.62, 114.79, 112.25, 100.41; HR-MS: for C_15_H_12_ClN_2_O^+^ [M+H]^+^ calculated 271.0633 *m/z*, found 271.0634 *m/z*.

*7-Chloro-4-[(4-hydroxyphenyl)amino]quinolinium chloride* (**2c**) [61]. Yield 99%; Mp. 303–305 °C; IR (Zn/Se ATR, cm^−1^): 3007w, 2768w, 1607m, 1586m, 1536m, 1506s, 1438m, 1358w, 1220m, 1161w, 1123w, 1096w, 893w, 847w, 809s, 784w, 727m, 658m; ^1^H-NMR (DMSO-*d*_6_), δ: 11.03 (br. s, 1H), 9.97 (br. s, 1H), 8.84 (d, *J =* 9.3 Hz, 1H), 8.44 (d, *J =* 7.0 Hz, 1H), 8.14 (br. s, 1H), 7.78 (br. s, 1H), 7.24 (d, *J =* 8.5 Hz, 2H), 6.95 (d, *J =* 8.8 Hz, 2H), 6.60 (d, *J =* 7.0 Hz, 1H); ^13^C-NMR APT (DMSO-*d*_6_), δ: 157.04, 155.21, 143.12, 139.19, 138.13, 127.75, 127.11, 126.09, 121.68, 119.27, 116.44, 115.67, 99.93; HR-MS: for C_15_H_12_ClN_2_O^+^ [M+H]^+^ calculated 271.0633 *m/z*, found 271.0636 *m/z*.

*7-Chloro-4-[(2-methoxyphenyl)amino]quinolinium chloride* (**3a**). Yield 31%; Mp. 230–231 °C; IR (Zn/Se ATR, cm^−1^): 2679w, 1611m, 1590m, 1540m, 1494w, 1452m, 1371w, 1279w, 1259s, 1214m, 1177w, 1114w, 1023m, 936w, 909w, 864w, 810s, 780s, 759m, 694m, 660m; ^1^H-NMR (DMSO-*d*_6_), δ: 11.16 (br. s, 1H), 8.98 (d, *J =* 9.0 Hz, 1H), 8.47 (d, *J =* 6.8, 1H), 8.23 (s, 1H), 7.80 (d, *J =* 9.0 Hz, 1H), 7.47–7.39 (m, 2H), 7.26 (d, *J =* 8.3 Hz, 1H), 7.10 (t, *J =* 7.5 Hz, 1H), 6.27 (d, *J =* 7.0 Hz, 1H), 3.75 (s, 3H); ^13^C-NMR APT (DMSO-*d*_6_), δ: 155.24, 154.23, 142.66, 138.88, 138.18, 129.70, 128.10, 127.17, 126.33, 124.64, 121.18, 119.06, 115.47, 112.87, 100.82, 55.72; HR-MS: for C_16_H_14_ClN_2_O^+^ [M+H]^+^ calculated 285.0789 *m/z*, found 285.0787 *m/z*.

*7-Chloro-4-[(3-methoxyphenyl)amino]quinolinium chloride* (**3b**). Yield 54%; Mp. 249–250 °C; IR (Zn/Se ATR, cm^−1^): 2647w, 1606m, 1584w, 1536w, 1501, 1441m, 1231m, 1208w, 1160w, 1096w, 1031m, 893m, 844w, 806s, 778s, 753w, 661s; ^1^H-NMR (DMSO-*d*_6_), δ: 11.34 (br. s, 1H), 8.98 (d, *J* *=* 9.0 Hz, 1H), 8.50 (d, *J =* 7.0 Hz, 1H), 8.20 (d, *J =* 2.0 Hz, 1H), 7.79 (dd, *J =* 9.0 Hz, *J =* 2.0 Hz, 1H), 7.45 (t, *J =* 8.0 Hz, 1 H), 7.06–7.04 (m, 2H), 6.99–6.96 (m, 1H), 6.83 (d, *J =* 7.0 Hz, 1H), 3.76 (s, 3H); ^13^C-NMR APT (DMSO-*d*_6_), δ: 160.28, 154.79, 143.16, 139.00, 138.28, 138.13, 130.67, 127.19, 126.41, 119.02, 117.26, 115.92, 113.09, 111.08, 100.49, 55.41; HR-MS: for C_16_H_14_ClN_2_O^+^ [M+H]^+^ calculated 285.0789 *m/z*, found 285.0788 *m/z*.

*7-Chloro-4-[(4-methoxyphenyl)amino]quinolinium chloride* (**3c**) [62]. Yield 44%; Mp. 286–287 °C; IR (Zn/Se ATR, cm^−1^): 2647w, 1606s, 1584m, 1536s, 1501s, 1442s, 1362w, 1231m, 1208m, 1160m, 1096w, 1031m, 893m, 845w, 806s, 778s, 753w; ^1^H-NMR (DMSO-*d*_6_), δ: 11.26 (s, 1H), 8.94 (d, *J* *=* 9.3 Hz, 1H), 8.46 (d, *J =* 7.0 Hz, 1H), 8.18 (d, *J =* 2.0 Hz, 1H), 7.79 (dd, *J =* 9.0 Hz, *J =* 2.3 Hz, 1H), 7.40–7.36 (m, 2H), 7.11–7.07 (m, 2H), 6.62 (d, *J =* 7.0 Hz, 1H), 3.80 (s, 3H); ^13^C-NMR APT (DMSO-*d*_6_), δ: 158.42, 155.24, 142.94, 138.96, 138.23, 129.42, 127.11 (2C), 126.31, 118.99, 115.70, 115.07, 99.93, 55.46; HR-MS: for C_16_H_14_ClN_2_O^+^ [M+H]^+^ calculated 285.0789 *m/z*, found 285.0788 *m/z*.

*7-Chloro-4-[(2-methylphenyl)amino]quinolinium chloride* (**4a**). Yield 93%; Mp. 247–248 °C; IR (Zn/Se ATR, cm^−1^): 2583w, 1605m, 1540m, 1488w, 1443w, 1359m, 1200m, 882w, 815s, 761w, 739s, 692s, 659m; ^1^H-NMR (DMSO-*d*_6_), δ: 11.28 (br. s, 1H), 9.01 (d, *J =* 9.3 Hz, 1H), 8.47 (d, *J =* 7.0 Hz, 1H), 8.22 (d, *J =* 1.8 Hz, 1H), 7.85 (dd, *J =* 9.2 Hz, *J =* 1.9 Hz, 1H), 7.48–7.46 (m, 1H), 7.42–7.35 (m, 3H), 6.21 (d, *J =* 7.0 Hz, 1H); 2.21 (s, 3H); ^13^C-NMR APT (DMSO-*d*_6_), δ: 155.50, 143.20, 139.00, 138.34, 135.34, 135.23, 131.60, 128.66, 127.63, 127.55, 127.28, 126.36, 119.13, 115.54, 99.93, 17.34; HR-MS: for C_16_H_14_ClN_2_^+^ [M+H]^+^ calculated 269.0840 *m/z*, found 269.0841 *m/z*.

*7-Chloro-4-[(3-methylphenyl)amino]quinolinium chloride* (**4b**). Yield 50%; Mp. 279–281 °C; IR (Zn/Se ATR, cm^−1^): 2646w, 1610m, 1578m, 1535m, 1446m, 1361w, 1326w, 1235w, 1208w, 1158w, 1095w, 895s, 850w, 802w, 768s, 685s; ^1^H-NMR (DMSO-*d*_6_), δ: 11.21 (br. s, 1H), 8.91 (d, *J =* 9.0 Hz, 1H), 8.5 (d, *J =* 7.0 Hz, 1H), 8.20 (d, *J =* 2.0 Hz, 1H), 7.85 (dd, *J =* 9.0 Hz, *J =* 2.0 Hz, 1H), 7.47–7.43 (m, 1H), 7.30–7.24 (m, 3H), 6.77 (d, *J =* 7.0 Hz, 1H), 2.38 (s, 3H); ^13^C-NMR APT (DMSO-*d*_6_), δ: 154.88, 143.27, 139.60, 139.08, 138.34, 136.86, 129.78, 128.30, 127.30, 126.21, 125.84, 122.48, 119.18, 115.89, 100.28, 20.93; HR-MS: for C_16_H_14_ClN_2_^+^ [M+H]^+^ calculated 269.0840 *m/z*, found 269.0848 *m/z*.

*7-Chloro-4-[(4-methylphenyl)amino]quinolinium chloride* (**4c**). Yield 90%; Mp. 297–298 °C; IR (Zn/Se ATR, cm^−1^): 2644w, 1605m, 1585m, 1536m, 1508m, 1444s, 1361w, 1325w, 1233w, 1211m, 1164w, 1098w, 896m, 864w, 806s, 778s, 676s; ^1^H-NMR (DMSO-*d*_6_), δ: 11.12 (s, 1H), 8.85 (d, *J* *=* 9.0 Hz, 1H), 8.49 (d, *J =* 7.0 Hz, 1H), 8.16 (d, *J =* 2.0 Hz, 1H), 7.87 (dd, *J =* 9.0 Hz, *J =* 2.0 Hz, 1H), 7.40–7.35 (m, 4H), 6.72 (d, *J =* 7.0 Hz, 1H), 2.38 (s, 3H); ^13^C-NMR APT (DMSO-*d*_6_), δ: 155.03, 143.29, 139.07, 138.34, 137.24, 134.28, 130.45, 127.30, 126.07, 125.42, 119.23, 115.84, 100.14, 20.71; HR-MS: for C_16_H_14_ClN_2_^+^ [M+H]^+^ calculated 269.0840 *m/z*, found 269.0838 *m/z*.

*7-Chloro-4-[(2-fluorophenyl)amino]quinolinium chloride* (**5a**). Yield 99%; Mp. 279–280 °C; IR (Zn/Se ATR, cm^−1^): 2580w, 1587m, 1540m, 1496m, 1145m, 1362w, 1239w, 1205m, 895m, 807s, 747s, 678s; ^1^H-NMR (DMSO-*d*_6_), δ: 11.32 (br. s, 1H), 8.98 (d, *J =* 9.2 Hz, 1H), 8.58 (d, *J =* 6.6 Hz, 1H), 8.25 (d, *J =* 2.1 Hz, 1H), 7.89 (dd, *J =* 8.1 Hz, *J =* 2.0 Hz, 1H), 7.62–7.48 (m, 3H), 7.44–7.39 (m, 1H), 6.49 (dd, *J =* 7.1 Hz, *J =* 2.6 Hz, 1H); ^13^C-NMR APT (DMSO-*d*_6_), δ: 156.82 (d, ^1^*J*_CF_ = 249.5 Hz), 155.19, 143.59, 138.96, 138.50, 130.18 (d, ^3^*J*_CF_ = 8.0 Hz), 128.96, 127.65, 126.29, 125.85, 124.29 (d, ^2^*J*_CF_ = 13.1 Hz), 119.30, 117.12 (d, ^2^*J*_CF_ = 19.1 Hz), 115.73, 100.78; HR-MS: for C_15_H_11_ClFN_2_^+^ [M+H]^+^ calculated 273.0589 *m/z*, found 273.0591 *m/z*.

*7-Chloro-4-[(3-fluorophenyl)amino]quinolinium chloride* (**5b**). Yield 48%; Mp. 241–242 °C; IR (Zn/Se ATR, cm^−1^): 2587w, 1580m, 1535w, 1482m, 1447m, 1404w, 1366w, 1231w, 1205w, 1142w, 879m, 825w, 778s, 720m, 681s; ^1^H-NMR (DMSO-*d*_6_), δ: 11.46 (br. s, 1H), 9.00 (d, *J =* 9.3 Hz, 1H), 8.55 (d, *J =* 7.0 Hz, 1H), 8.21 (d, *J =* 2.3 Hz, 1H), 7.81 (dd, *J =* 9.2 Hz, *J =* 2.1 Hz, 1H), 7.61–7.55 (m, 1H), 7.43–7.38 (m, 2H), 7.25 (td, *J =* 8.4 Hz, *J =* 2.3 Hz, 1H), 6.90 (d, *J =* 6.8 Hz, 1H); ^13^C-NMR APT (DMSO-*d*_6_), δ: 162.50 (d, ^1^*J*_CF_ = 245.5 Hz), 154.59, 143.45, 139.01, 138.85 (d, ^3^*J*_CF_ = 10.1 Hz), 138.39, 131.52 (d, ^3^*J*_CF_ = 9.1 Hz), 127.37, 126.49, 121.29 (d, ^4^*J*_CF_ = 3.0 Hz), 119.09, 116.08, 114.17 (d, ^2^*J*_CF_ = 20.1 Hz), 112.48 (d, ^2^*J*_CF_ = 20.1 Hz), 100.70; HR-MS: for C_15_H_11_ClFN_2_^+^ [M+H]^+^ calculated 273.0589 *m/z*, found 273.0593 *m/z*.

*7-Chloro-4-[(4-fluorophenyl)amino]quinolinium chloride* (**5c**). Yield 54%; Mp. 304–305 °C; IR (Zn/Se ATR, cm^−1^): 2646w, 1606m, 1579w, 1535w, 1498m, 1445m, 1361w, 1205s, 1093w, 894w, 847m, 824s, 804s, 782s, 757m, 681s; ^1^H-NMR (DMSO-*d*_6_), δ: 11.18 (br. s, 1H), 8.86 (d, *J =* 9.0 Hz, 1H), 8.53 (d, *J =* 6.8 Hz, 1H), 8.17 (d, *J =* 2.3 Hz, 1H), 7.89 (dd, *J =* 9.0 Hz, *J =* 2.3 Hz, 1H), 7.54 (dd, *J* *=* 9.0 Hz, *J =* 5.0 Hz, 2H), 7.45–7.41 (m, 2H), 6.72 (d, *J =* 7.0 Hz, 1H); ^13^C-NMR APT (DMSO-*d*_6_), δ: 160.93 (d, ^1^*J*_CF_ = 244.5 Hz), 155.19, 143.48, 139.05, 138.42, 133.27, 127.97 (d, ^3^*J*_CF_ = 9.1 Hz), 127.41, 126.11, 119.27, 116.89 (d, ^2^*J*_CF_ = 22.1 Hz), 115.86, 100.23; HR-MS: for C_15_H_11_ClFN_2_^+^ [M+H]^+^ calculated 273.0589 *m/z*, found 273.0594 *m/z*.

*7-Chloro-4-[(2-chlororophenyl)amino]quinolinium chloride* (**6a**). Yield 99%; Mp. 241–243 °C; IR (Zn/Se ATR, cm^−1^): 2733w, 1606m, 1578w, 1535s, 1474w, 1439m, 1358w, 1205w, 1040w, 882w, 805s, 757s; ^1^H-NMR APT (DMSO-*d*_6_), δ: 11.52 (br. s, 1H), 9.02–8.99 (m, 1H), 8.55 (m, 1H), 8.27 (br. s, 1H), 7.87 (d, *J =* 9.0 Hz, 1H), 7.73 (d, *J =* 7.0 Hz, 1H), 7.63–7.52 (m, 3H), 6.28–6.24 (m, 1H); ^13^C-NMR (DMSO-*d*_6_), δ: 155.48, 143.60, 139.17, 138.72, 134.18, 131.30, 130.99, 130.48, 130.04, 129.26, 127.81, 126.52, 119.45, 115.73, 100.90; HR-MS: for C_15_H_11_Cl_2_N_2_^+^ [M+H]^+^ calculated 289.0294 *m/z*, found 289.0297 *m/z*.

*7-Chloro-4-[(3-chlororophenyl)amino]quinolinium chloride* (**6b**). Yield 50%; Mp. 261–262 °C; IR (Zn/Se ATR, cm^−1^): 2645 w, 1611w, 1579s, 1530m, 1445s, 1404w, 1356m, 1330w, 1239w, 1207m, 1164w, 1089m, 881m, 825m, 796s, 771s, 720s, 696s, 673s; ^1^H-NMR (DMSO-*d*_6_), δ: 11.38 (br. s, 1H), 8.95 (d, *J =* 9.0 Hz, 1H), 8.56 (d, *J =* 7.0 Hz, 1H), 8.22 (d, *J =* 2.0 Hz, 1H), 7.87 (dd, *J =* 9.0 Hz, *J* *=* 2.0 Hz, 1H), 7.63–7.59 (m, 2H), 7.51–7.48 (m, 2H), 6.88 (d, *J =* 7.0 Hz, 1H); ^13^C-NMR APT (DMSO-*d*_6_), δ: 154.68, 143.60, 139.08, 138.69, 138.45, 133.96, 131.52, 127.48, 127.33, 126.38, 125.20, 124.00, 119.23, 116.13, 100.73; HR‑MS: for C_15_H_11_Cl_2_N_2_^+^ [M+H]^+^ calculated 289.0294 *m/z*, found 289.0296 *m/z*.

*7-Chloro-4-[(4-chlororophenyl)amino]quinolinium chloride* (**6c**) [63]. Yield 51%; Mp. 292–293 °C; IR (Zn/Se ATR, cm^−1^): 2645w, 1608m, 1581w, 1482w, 1441m, 1360w, 1232w, 1207w, 1084w, 894m, 846w, 806s, 772m, 670s; ^1^H-NMR (DMSO-*d*_6_), δ: 11.25 (br. s, 1H), 8.89 (d, *J =* 9.0 Hz, 1H), 8.55 (d, *J =* 6.8 Hz, 1H), 8.19 (d, *J =* 2.0 Hz, 1H), 7.88 (dd, *J =* 9.0 Hz, *J =* 2.0 Hz, 1H), 7.65–7.62 (m, 2H), 7.55–7.52 (m, 2H), 6.84 (d, *J =* 7.0 Hz, 1 H); ^13^C-NMR APT (DMSO-*d*_6_), δ: 154.79, 143.57, 139.11, 138.45, 136.05, 131.65, 129.94, 127.46, 127.25, 126.23, 119.29, 116.08, 100.51; HR-MS: for C_15_H_11_Cl_2_N_2_^+^ [M+H]^+^ calculated 289.0294 *m/z*, found 289.0296 *m/z*.

*4-[(2-Bromophenyl)amino]-7-chloroquinolinium chloride* (**7a**). Yield 83%; Mp. 234–235 °C; IR (Zn/Se ATR, cm^−1^): 2730w, 1606m, 1579m, 1535s, 1470w, 1440m, 1360w, 1231w, 1209m, 1156w, 1034w, 883w, 806s, 756s, 653s; ^1^H-NMR (DMSO-*d*_6_), δ: 11.52 (br. s, 1H), 9.00 (d, *J =* 9.3 Hz, 1H), 8.55 (d, *J =* 7.0 Hz, 1H), 8.27 (d, *J =* 2.0 Hz, 1H), 7.90–7.86 (m, 2H), 7.61–7.60 (m, 2H), 7.46 (ddd, *J* *=* 8.2 Hz, *J =* 5.3 Hz, *J =* 3.9 Hz, 1H), 6.23 (d, *J =* 7.0 Hz, 1H); ^13^C-NMR APT (DMSO-*d*_6_), δ: 155.30, 143.32, 138.95, 138.54, 135.49, 133.95, 130.57, 130.01, 129.69, 127.63, 126.27, 121.84, 119.24, 115.46, 100.67; HR-MS: for C_15_H_11_BrClN_2_^+^ [M+H]^+^ calculated 332.9789 *m/z*, found 332.9792 *m/z*.

*4-[(3-Bromophenyl)amino]-7-chloroquinolinium chloride* (**7b**). Yield 97%; Mp. 294–295 °C; IR (Zn/Se ATR, cm^−1^): 2731w, 1609m, 1574m, 1528m, 1446m, 1407w, 1360w, 1323w, 1231w, 1207w, 1158w, 1096w, 1031m, 893m, 844w, 806s, 778s, 753w, 661s; ^1^H-NMR (DMSO-*d*_6_), δ: 11.37 (br. s, 1H), 8.93 (d, *J =* 9.0 Hz, 1H), 8.57 (d, *J =* 7.0 Hz, 1H), 8.21 (d, *J =* 2.0 Hz, 1H), 7.87 (dd, *J =* 9.0 Hz, *J =* 2.0 Hz, 1H), 7.76–7.75 (m, 1H), 7.64–7.61 (m, 1H), 7.55–7.52 (m, 2H), 6.88 (d, *J =* 7.0 Hz, 1H); ^13^C-NMR APT (DMSO-*d*_6_), δ: 154.88, 143.61, 139.08, 138.80, 138.45, 131.76, 130.23, 128.01, 127.48, 126.34, 124.37, 122.27, 119.23, 116.12, 100.71; HR-MS: for C_15_H_11_BrClN_2_^+^ [M+H]^+^ calculated 332.9789 *m/z*, found 332.9793 *m/z*.

*4-[(4-Bromophenyl)amino]-7-chloroquinolinium chloride* (**7c**). Yield 99%; Mp. 293–294 °C; IR (Zn/Se ATR, cm^−1^): 2699w, 1606m, 1578w, 1529m, 1480w, 1440m, 1360w, 1232w, 1208w, 1063w, 894m, 806s, 769m, 665m; ^1^H-NMR (DMSO-*d*_6_), δ: 11.18 (br. s, 1H), 8.85 (d, *J =* 9.3 Hz, 1H), 8.55 (d, *J =* 8.0 Hz, 1 H), 8.17 (s, 1H), 7.89 (d, *J =* 9.0 Hz, 1H), 7.77 (d, *J =* 8.3 Hz, 2H), 7.47 (d, *J =* 8.3 Hz, 2H), 6.86 (d, *J =* 7.0 Hz, 1H); ^13^C-NMR APT (DMSO-*d*_6_), δ: 154.68, 143.69, 139.14, 138.46, 136.49, 132.89, 128.08, 127.49, 126.15, 120.01, 119.36, 116.11, 100.57; HR-MS: for C_15_H_11_BrClN_2_^+^ [M+H]^+^ calculated 332.9789 *m/z*, found 332.9794 *m/z*.

*7-Chloro-4-[(2-trifluoromethylphenyl)amino]quinolinium chloride* (**8a**). Yield 59%; Mp. 217–218 °C; IR (Zn/Se ATR, cm^−1^): 2382w, 1584m, 1538m, 1494w, 1441m, 1420w, 1353w, 1309s, 1235w, 1206m, 1168m, 1124m, 1090m, 1000w, 954w, 910w, 864m, 812s, 772m, 730w; ^1^H-NMR (DMSO-*d*_6_), δ: 11.44 (br. s, 1H), 8.98 (d, *J =* 9.0 Hz, 1H), 8.53 (d, *J =* 6.8 Hz, 1H), 8.27-8.26 (m, 1H), 7.85 (dd, *J* *=* 9.0 Hz, *J =* 2.0 Hz, 1H), 7.78–7.70 (m, 2H), 7.55–7.52 (m, 2H), 6.16 (d, *J =* 7.0 Hz, 1H); ^13^C‑NMR APT (DMSO-*d*_6_), δ: 156.54, 147.49, 139.37, 137.50, 134.97, 134.80 (q, ^3^*J*_CF_ = 1.5 Hz), 129.83, 127.83 (q, ^3^*J*_CF_ = 4.4 Hz), 127.74, 127.19 (q, ^2^*J*_CF_ = 30.1 Hz), 126.42, 126.12, 125.88, 123.17 (q, ^1^*J*_CF_ = 273.6 Hz), 115.43, 100.92; HR-MS: for C_16_H_11_ClF_3_N_2_^+^ [M+H]^+^ calculated 323.0557 *m/z*, found 323.0561 *m/z*.

*7-Chloro-4-[(3-trifluoromethylphenyl)amino]quinolinium chloride* (**8b**). Yield 49%; Mp. 291–292 °C; IR (Zn/Se ATR, cm^−1^): 2744w, 1608m, 1580m, 1535m, 1452m, 1411w, 1371w, 1324s, 1267w, 1208w, 1168m, 1104m, 1062m, 963w, 890s, 808s, 772m, 697m, 667s; ^1^H-NMR (DMSO-*d*_6_), δ: 11.42 (br. s, 1H), 8.93 (d, *J =* 9.0 Hz, 1H), 8.59 (d, *J =* 7.0 Hz, 1H), 8.22 (d, *J =* 2.0 Hz, 1H), 7.92–7.89 (m, 2H), 7.87–7.77 (m, 3H), 6.90 (d, *J =* 7.0 Hz, 1H); ^13^C-NMR APT (DMSO-*d*_6_), δ: 154.70, 143.84, 139.17, 138.50, 138.15, 131.21, 130.56 (q, ^2^*J*_CF_ = 32.2 Hz), 129.27, 127.58, 126.31, 123.90 (q, ^3^*J*_CF_ = 4.0 Hz), 123.76 (q, ^1^*J*_CF_ = 273.7 Hz), 122.03 (q, ^3^*J*_CF_ = 4.0 Hz), 119.35, 116.25, 100.69; HR-MS: for C_16_H_11_ClF_3_N_2_^+^ [M+H]^+^ calculated 323.0557 *m/z*, found 323.0560 *m/z*.

*7-Chloro-4-[(4-trifluoromethylphenyl)amino]quinolinium chloride* (**8c**). Yield 99%; Mp. 301–302 °C; IR (Zn/Se ATR, cm^−1^): 2646w, 1582m, 1528m, 1442m, 1411w, 1319s, 1234w, 1207w, 1156s, 1104s, 1063m, 1008w, 965w, 898m, 854m, 809s, 779w, 756w, 666s; ^1^H-NMR (DMSO-*d*_6_), δ: 11.37 (br. s, 1H), 8.93 (d, *J =* 9.0 Hz, 1H), 8.61 (d, *J =* 7.0 Hz, 1H), 8.21 (d, *J =* 2.0 Hz, 1H), 7.94–7.90 (m, 3H), 7.75 (d, *J =* 8.5 Hz, 2H), 7.04 (d, *J* = 7.0 Hz, 1H); ^13^C-NMR APT (DMSO-*d*_6_), δ: 154.31, 143.99, 141.18, 139.84 (q, ^1^*J*_CF_ = 268.6 Hz), 139.32, 138.51, 127.62, 127.05 (q, ^2^*J*_CF_ = 32.2 Hz), 127.03 (q, ^3^*J*_CF_= 4.0 Hz), 126.33, 125.36, 119.48, 116.47, 101.14; HR-MS: for C_16_H_11_ClF_3_N_2_^+^ [M+H]^+^ calculated 323.0557 *m/z*, found 323.0559 *m/z*.

*7-Chloro-4-[(2-nitrophenyl)amino]quinolinium chloride* (**9a**). Yield 58%; Mp. 219–220 °C; IR (Zn/Se ATR, cm^−1^): 2350w, 1629w, 1583s, 1537m, 1470w, 1440m, 1420m, 1394w, 1351m, 1303s, 1204m, 1088m, 999w, 863m, 812s, 743m; ^1^H-NMR (DMSO-*d*_6_), δ: 11.63 (br. s, 1H), 8.94 (d, *J =* 9.0 Hz, 1H), 8.59 (d, *J =* 6.8 Hz, 1H), 8.16 (d, *J =* 8.8 Hz, 2H), 7.57 (d, *J =* 2.0 Hz, 2H), 6.88 (d, *J =* 7.0 Hz, 2H), 6.59 (d, *J =* 6.8 Hz, 1H); ^13^C-NMR APT (DMSO-*d*_6_), δ: 155.32, 145.36, 143.32, 138.92, 137.69, 135.32, 130.55, 130.09, 129.55, 127.81, 126.32, 126.22, 118.28, 115.93, 100.84; HR-MS: for C_15_H_11_ClN_3_O_2_^+^ [M+H]^+^ calculated 300.0534 *m/z*, found 300.0537 *m/z*.

*7-Chloro-4-[(3-nitrophenyl)amino]quinolinium chloride* (**9b**). Yield 53%; Mp. 273–274 °C; IR (Zn/Se ATR, cm^−1^): 2637w, 1585m, 1535m, 1515s, 1442s, 1407w, 1352s, 1332m, 1235m, 1208m, 1169w, 1092m, 908w, 850w, 811s, 775w, 735s, 718s, 687s; ^1^H-NMR (DMSO-*d*_6_), δ: 11.38 (br.s, 1H), 8.88 (d, *J =* 9.3 Hz, 1H), 8.63 (d, *J =* 7.0 Hz, 1H), 8.36 (t, *J =* 2.0 Hz 1H), 8.24 (ddd, *J =* 8.2 Hz, *J =* 2.3 Hz, *J* *=* 1.0 Hz, 1H), 8.19 (d, *J =* 2.0 Hz, 1H), 8.00–7.98 (m, 1H), 7.92 (dd, *J =* 9.2 Hz, *J =* 2.1 Hz, 1H), 7.85 (t, *J =* 8.0 Hz, 1H), 7.05 (d, *J =* 6.8 Hz, 1H); ^13^C-NMR APT (DMSO-*d*_6_), δ: 154.59, 148.68, 144.13, 139.28, 138.62, 138.59, 131.46, 131.40 127.75, 126.27, 121.81, 119.91, 119.52, 116.44, 101.02; HR-MS: for C_15_H_11_ClN_3_O_2_^+^ [M+ H]^+^ calculated 300.0534 *m/z*, found 300.0536 *m/z*.

*7-Chloro-4-[(4-nitrophenyl)amino]quinolinium chloride* (**9c**). Yield 39%; Mp. 286–287 °C; IR (Zn/Se ATR, cm^−1^): 2645w, 1618m, 1583s, 1543m, 1504m, 1450m, 1377w, 1343s, 1297m, 1238m, 1211m, 1178w, 1105m, 1095m, 996w, 917w, 854m, 832m, 803w, 744w, 694w; ^1^H-NMR (DMSO-*d*_6_), δ: 11.36 (br. s, 1H), 8.87 (d, *J =* 9.0 Hz, 1H), 8.70 (d, *J =* 6.8 Hz, 1H), 8.40–8.36 (m, 1H), 8.19 (d, *J =* 2.3 Hz, 1H), 7.95–7.90 (m, 1H), 7.79–7.77 (m, 2H), 7.24 (d, *J =* 7.0 Hz, 1H), 6.61–6.57 (m, 1H); ^13^C-NMR APT (DMSO-*d*_6_), δ: 155.80, 153.62, 144.65, 144.07, 139.73, 138.64, 127.90, 126.48, 125.50, 124.40, 119.89, 117.09, 102.37; HR-MS: for C_15_H_11_ClN_3_O_2_^+^ [M+H]^+^ calculated 300.0534 *m/z*, found 300.0537 *m/z*.

### 3.3. QSAR Study

Calculation of log *P* values and electronic Hammett’s σ parameters, both for the uncharged molecules, were carried out on the software ACD/Percepta ver. 2012 (Advanced Chemistry Development, Inc., Toronto, ON, Canada). Correlation and regression analyses of the QSAR study were run on a personal computer using the Microsoft Excel program. In the equations, the figures in the parentheses are the standard errors of the regression coefficients, *n* is the number of compounds, *r* is the correlation coefficient, *F* is the significance test (F-test) and *s* is the standard error of estimate. F‑test values are statistically significant for all equations at 1% level of probability.

### 3.4. Study of Inhibition of Photosynthetic Electron Transport (PET) in Spinach Chloroplasts

Chloroplasts were prepared from spinach (*Spinacia oleracea* L.) according to Masarovicova and Kralova [64]. The inhibition of photosynthetic electron transport (PET) in spinach chloroplasts was determined spectrophotometrically (Genesys 6, Thermo Scientific), using an artificial electron acceptor 2,6-dichlorophenol-indophenol (DCPIP) according to Kralova *et al.* [65], and the rate of photosynthetic electron transport was monitored as a photoreduction of DCPIP. The measurements were carried out in phosphate buffer (0.02 mol/L, pH 7.2) containing sucrose (0.4 mol/L), MgCl_2_ (0.005 mol/L) and NaCl (0.015 mol/L). The chlorophyll content was 30 mg/L in these experiments and the samples were irradiated (~100 W/m^2^ with 10 cm distance) with a halogen lamp (250 W) using a 4 cm water filter to prevent warming of the samples (suspension temperature 22 °C). The studied compounds were dissolved in DMSO due to their limited water solubility. The applied DMSO concentration (up to 4%) did not affect the photochemical activity in spinach chloroplasts. The inhibitory efficiency of the studied compounds was expressed by IC_50_ values, *i.e.*, by molar concentration of the compounds causing 50% decrease in the oxygen evolution rate relative to the untreated control. The comparable IC_50_ value for a selective herbicide 3-(3,4-dichlorophenyl)-1,1-dimethylurea, DCMU (Diurone^®^) was about 1.9 μmol/L. The results are summarized in Table 1.

### 3.5. Study of Chlorophyll a Fluorescence in Spinach Chloroplasts

The fluorescence emission spectra of chlorophyll *a* (Chl*a*) in spinach chloroplasts were recorded on fluorescence spectrophotometer F-2000 (Hitachi, Tokyo, Japan) using excitation wavelength λ_ex_ = 436 nm for monitoring fluorescence of Chl*a*, excitation slit 20 nm and emission slit 10 nm. The samples were kept in the dark for 2 min before measuring. The phosphate buffer used for dilution of the chloroplast suspension was the same as described above. Due to low aqueous solubility the compounds were added to chloroplast suspension in DMSO solution. The DMSO concentration in all samples was the same as in the control (10%). In fluorescence experiments the chlorophyll concentration in chloroplast suspension was kept constant, 10 mg/L.

### 3.6. In Vitro Antimycobacterial Evaluation

As antimycobacterial screening was performed in two different institutions, two different procedures were used for testing. The evaluation of *in vitro* antimycobacterial activity of the compounds was performed against *Mycobacterium marinum* CAMP 5644, *M. kansasii* DSM 44162 and *M. smegmatis* ATCC 700084. The broth dilution micro-method in Middlebrook 7H9 medium (Difco, Lawrence, KS, USA) supplemented with BD BBL™ Middlebrook ADC Enrichment (Becton, Dickinson & Co., Franklin Lakes, NJ, USA) was used to determine the minimum inhibitory concentration (MIC) as previously described [66]. The compounds were dissolved in DMSO (Sigma-Aldrich, Munich, Germany), and the final concentration of DMSO did not exceed 2.5% of the total solution composition. The final concentrations of the evaluated compounds ranging from 256 μg/mL to 0.125 μg/mL were obtained by twofold serial dilution of the stock solution in microtiter plate with sterile medium. Bacterial inocula were prepared by transferring colonies from culture to sterile water. The cell density was adjusted to 0.5 McFarland units (1.5 × 10^8^ cfu) using a densitometer (Densi-La-Meter, LIAP, Riga, Latvia). The final inoculum was made by 1:1000 dilution of the suspension with sterile water. Drug-free controls, sterility controls and controls consisted of medium and DMSO alone were included. The conditions of static incubation in the darkness in an aerobic atmosphere were as follows: 3 days at 37 °C for *M. smegmatis*, 7 days at 37 °C for *M. kansasii* and 21 days at 28 °C for *M.* *marinum*, as was described recently [45,46,67,68].

A clinical isolate of *M. avium* subsp. *paratuberculosis* CIT03 was grown in Middlebrook broth (MB), supplemented with Oleic-Albumin-Dextrose-Catalase supplement (OADC, Becton, Dickinson & Co.) and mycobactin J (2 µg/mL). Identification of this isolate was performed using biochemical and molecular protocols. At log phase growth, culture (10 mL) was centrifuged at 15,000rpm/20 min using a bench top centrifuge (Model CR 4-12, Jouan Inc., Winchester, VA, USA). Following removal of the supernatant, the pellet was washed in fresh Middlebrook 7H9GC broth and re-suspended in fresh supplemented MB (10 mL). The turbidity was adjusted to match McFarland standard No.1 (3 × 10^8^ cfu) with MB broth. A further 1:20 dilution of the culture was then performed in MB broth. The antimicrobial susceptibility of the mycobacterial species was investigated in a 96-well plate format. In these experiments, sterile deionised water (300 µL) was added to all outer-perimeter wells of the plates to minimize evaporation of the medium in the test wells during incubation. Each evaluated compound (100 µL) was incubated with each of the mycobacterial species (100 µL). Dilutions of each compound were prepared in duplicate. For all synthesized compounds, final concentrations ranged from 1,000 µg/mL to 8 µg/mL. All compounds were prepared in DMSO and subsequent dilutions were made in supplemented MB. The plates were sealed with parafilm and incubated at 37 °C, for 11 days in the case of *M. avium* subsp. *paratuberculosis*. Following incubation, a 10% addition of alamarBlue (AbD Serotec, Kidlington, UK) was mixed into each well and readings at 570 nm and 600 nm were taken, initially for background subtraction and subsequently after 24 h re-incubation. The background subtraction is necessary for strongly coloured compounds, where the colour may interfere with the interpretation of any colour change. For non-interfering compounds, a blue colour in the well was interpreted as an absence of growth and a pink colour was scored as growth. The minimum inhibitory concentrations (MICs) were initially defined as the lowest concentration which prevented a visual colour change from blue to pink, as was described recently [15,16,41,44,46].

The MICs were defined as the lowest concentration of the compound at which no visible bacterial growth was observed. The MIC value is routinely and widely used in bacterial assays and is a standard detection limit according to the Clinical and Laboratory Standards Institute (CLSI). Isoniazid, pyrazinamide, rifampicin and ciprofloxacin (Sigma-Aldrich, Munich, Germany) were used as reference antimycobacterial drug. The results are summarized in Table 1.

### 3.7. *In Vitro* Cytotoxicity Assay

Human monocytic leukemia THP-1 cells were obtained from the European Collection of Cell Cultures (ECACC, Salisbury, UK; Methods of characterization: DNA Fingerprinting (Multilocus probes) and isoenzyme analysis). These cells were routinely cultured in RPMI 1640 (Lonza, Verviers, Belgium) medium supplemented with 10% fetal bovine serum (FBS, Sigma-Aldrich, Munich, Germany), 2% l-glutamine, 1% penicillin and streptomycin (Lonza, Verviers, Belgium) at 37 °C with 5% CO_2_. Cells were passaged at approximately 1 week intervals. Cells were routinely tested for the absence of mycoplasma (Hoechst 33258 staining method). Cytotoxicity of the compounds was determined using a WST-1 assay kit (Roche Diagnostics, Mannheim, Germany) according to the manufacturer’s instructions. THP-1 cells were exposed for 24 h at 37 °C to various compound concentrations ranging from 1.25 μmol/L to 20 μmol/L in RPMI 1640 medium. For WST-1 assays, cells were seeded into 96-well plates (5 × 10^4^ cells·well^−1^ in 100 μL culture medium) in triplicate in serum-free RPMI 1640 medium and measurements were taken 24 h after the treatment with the compounds. The maximum concentration of DMSO in the assays never exceeded 0.1%. All data from three independent experiments were evaluated using GraphPad Prism 5.00 software (GraphPadSoftware, San Diego, CA, USA). The results are summarized in Table 1.

## 4. Conclusions

A series of twenty-five ring-substituted 4-arylamino-7-chloroquinolinium chlorides were prepared and characterized. The prepared compounds were tested for their ability to inhibit photosynthetic electron transport (PET) in spinach chloroplasts (*Spinacia oleracea* L.) and for their antimycobacterial activity against *Mycobacterium* *marinum*, *M. kansasii*, *M. smegmatis* and *M. avium* subsp. *paratuberculosis*. 7-Chloro-4-[(3-trifluoromethylphenyl)amino]quinolinium chloride (**8b**) showed the highest PET inhibition within the whole series and PET-inhibiting activity of *ortho*-substituted compounds was significantly lower than this of *meta*- and *para*-substituted ones. 4‑[(2‑Bromophenyl)amino]-7-chloroquinolinium chloride (**7a**) showed comparatively good biological activity against *M. marinum*, *M. kansasii*, *M. smegmatis* and 7-chloro-4-[(2-methylphenyl)amino] quinolinium chloride (**4a**) demonstrated quite good biological activity against *M.* *smegmatis* and *M. avium* subsp. *paratuberculosis*. Both compounds showed comparable or higher activity than the standard isoniazid. It can be stated that lipophilicity and especially electronic properties of aniline substituents influenced the biological activities of compounds. The dependences of antimycobacterial efficacy in particular on electronic properties showed bilinear trends with the σ optimum 0.22 for *M. marinum* and *M. kansasii*, and with the σ optimum −0.17 for *M. smegmatis* and *M.* *avium* subsp. *paratuberculosis*. None of the tested compounds were cytotoxic in the highest available concentration used in these cytotoxicity assays (LD_50_ > 20 μmol/L).
